# COVID-19 and pulmonary tuberculosis – A diagnostic dilemma

**DOI:** 10.1016/j.radcr.2021.07.079

**Published:** 2021-08-02

**Authors:** Som Subhro Biswas, Sandeep Singh Awal, Sampreet Kaur Awal

**Affiliations:** a Department of Radiology, Radiance Teleradiology Services, Navi Mumbai, India; b Department of Radiology, Jeevandeep Diagnostics, Jamshedpur, India; c Department of Microbiology, Guru Gobind Singh Medical College & Hospital, Faridkot, Punjab, India

**Keywords:** COVID -19, Tuberculosis, Co-infection, Ground glass opacities, HRCT, Case report, COVID-19, Coronavirus disease 2019, SARS-CoV-2, Severe acute respiratory syndrome coronavirus 2, TB, Tuberculosis, CT, Computed tomography, HRCT, High resolution computed tomography, AFB, Acid-fast bacilli, RT-PCR, Reverse transcriptase-polymerase chain reaction, ICU, Intensive care unit, CO-RADS, COVID-19 Reporting and Data System, GGOs, Ground glass opacities, DOTS, Directly Observed Therapy, Short-Course, CRP, C-reactive protein, WBC, White blood cell, DNA, Deoxyribonucleic acid

## Abstract

Coronavirus disease 2019 (COVID-19) is an infectious disease caused by the severe acute respiratory syndrome coronavirus 2 (SARS-CoV-2). Meanwhile, pulmonary tuberculosis(TB) is one of the most common infective lung diseases in developing nations. The concurrence of pulmonary TB and COVID-19 can lead to poor prognosis, owing to the pre-existing lung damage caused by TB. Case presentation: We describe the imaging findings in 3 cases of COVID-19 pneumonia with co-existing pulmonary TB on HRCT thorax. The concurrence of COVID-19 and pulmonary TB can be a diagnostic dilemma. Correct diagnosis and prompt management is imperative to reduce mortality and morbidity. Hence it is pertinent for imaging departments to identify and report these distinct entities when presenting in conjunction.

## Background

Coronavirus disease 2019 (COVID-19) is an infectious disease caused by the severe acute respiratory syndrome coronavirus 2 (SARS-CoV-2) with the first human case reported in December 2019 [1,2,3]. COVID-19 predominantly causes respiratory illness, ranging from mild to severe pneumonia [3,4]. Patients with pre-existing pulmonary tuberculosis (TB) can have a higher risk of COVID-19 infection [4]. The concurrence of pulmonary TB and COVID-19 can lead to poor prognosis, owing to the pre-existing lung damage caused by TB [4]. India has a high case burden of TB, accounting for a quarter of the TB cases worldwide [4,5]. Patients with co-existing COVID-19 and pulmonary TB can be a diagnostic challenge [6,7]. Correct diagnosis and prompt management is imperative to reduce mortality and morbidity caused by the concurrence of pulmonary TB and COVID-19. Hence it is pertinent for imaging departments to identify and report these distinct entities when presenting in conjunction.

Computed tomography (CT) of the thorax is a useful aid in detecting and characterizing the lesions of pulmonary TB as well as monitoring late complications [5]. It can correctly diagnose cavitation, bronchiectasis, miliary opacities, lung nodules, fibrotic changes as well as mediastinal and hilar lymphadenopathy [5,8]. Further, high resolution CT thorax (HRCT thorax) can detect and delineate the extent of ground glass opacities, consolidation and/or fibrosis caused by COVID-19 [3].

We present the imaging findings in COVID-19 with concurrent pulmonary TB in 3 patients without any prior history of mycobacterial pulmonary infection. HRCT Thorax was done using 32 slice Multidetector CT machine using thin sections (1 mm slice thickness). Final diagnosis of pulmonary TB was made by demonstrating acid-fast bacilli (AFB) on sputum microscopy, as per the current guidelines [5]. Confirmation of COVID-19 was made based on Reverse transcriptase-polymerase chain reaction (RT-PCR) from nasopharyngeal swab [9].

## Case 1

A 70-year-old male patient presented with fever, chronic cough with exacerbation of symptoms and severe breathlessness since 1 week. Patient also had history of chest pain and few episodes of hemoptysis. On admission, HRCT thorax & RT-PCR for COVID-19 were performed.

**Fig. 1:**
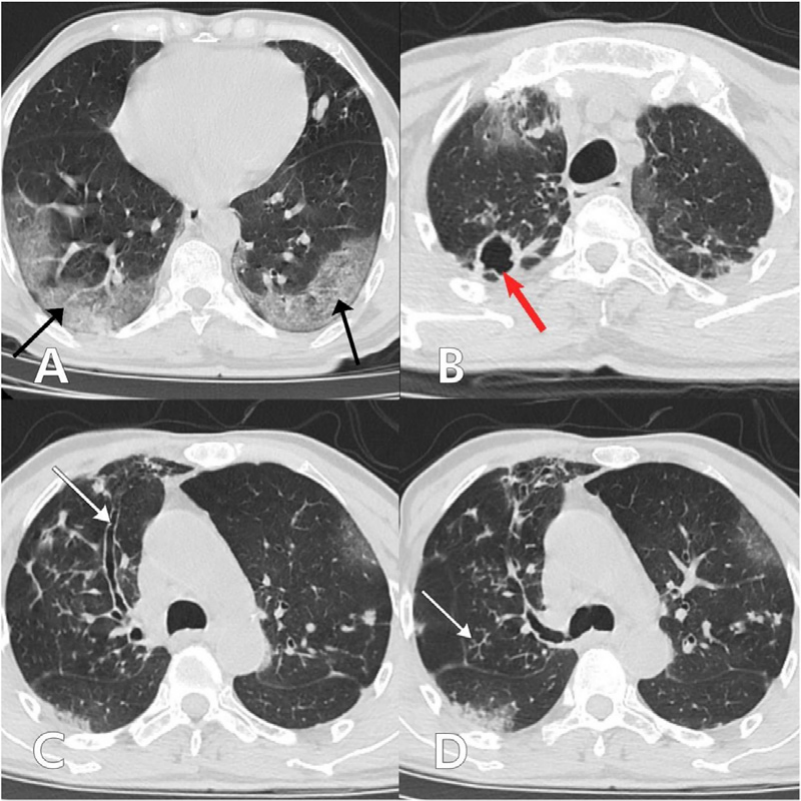
A-D: Axial HRCT thorax showing (A) Peripheral subpleural GGOs (black arrows) with superimposed septal thickening (“crazy paving pattern”) in lower lobes of bilateral lung parenchyma. (B) Cavitatory lesion (red arrow) in right upper lobe with associated fibrotic changes and superimposed ground glass opacities in bilateral upper lobes. (C) Cylindrical bronchiectasis involving right upper lobe (white arrow) with peripheral subpleural GGO in left upper lobe and superior segment of right lower lobe. (D) Centrilobular nodules with “tree-in-bud” appearance in right upper lobe (white arrow) (Color version of figure is available online).

HRCT thorax revealed multifocal subpleural ground glass opacities with superimposed septal thickening (“crazy paving pattern”) in bilateral lung parenchyma, with dorsal and lower lobe predominance (Fig. 1A). Chest CT Severity Score was 9 out of 25. Above findings were in favour of atypical pneumonia with CO-RADS (COVID-19 Reporting and Data System) category 5, typical for COVID-19 [10]. Further, fibro-cavitatory changes with cylindrical bronchiectasis involving bilateral upper lobes were seen, more in right upper lobe of lung (Fig. 1B,C). Multiple centrilobular nodules and “tree-in-bud” branching opacities were also noted in right upper lobe and in superior segments of bilateral lower lobes of lung (Fig. 1D). These features were suspicious for concurrent pulmonary tuberculosis, based on the clinical features and imaging [5,11]. Reverse transcriptase-polymerase chain reaction (RT-PCR) from nasopharyngeal swab came positive for COVID-19. Furthermore, sputum microscopy revealed acid-fast bacilli (AFB), suggesting pulmonary tuberculosis.

Patient was shifted to ICU on day 3 due to worsening dyspnoea. Despite intensive care and oxygen support, patient developed multiorgan dysfunction and succumbed on day 5.

## Case 2

A 67-year-old male patient presented with acute on chronic cough, fever and generalized weakness. HRCT thorax & RT-PCR for COVID-19 were performed.

**Fig. 2:**
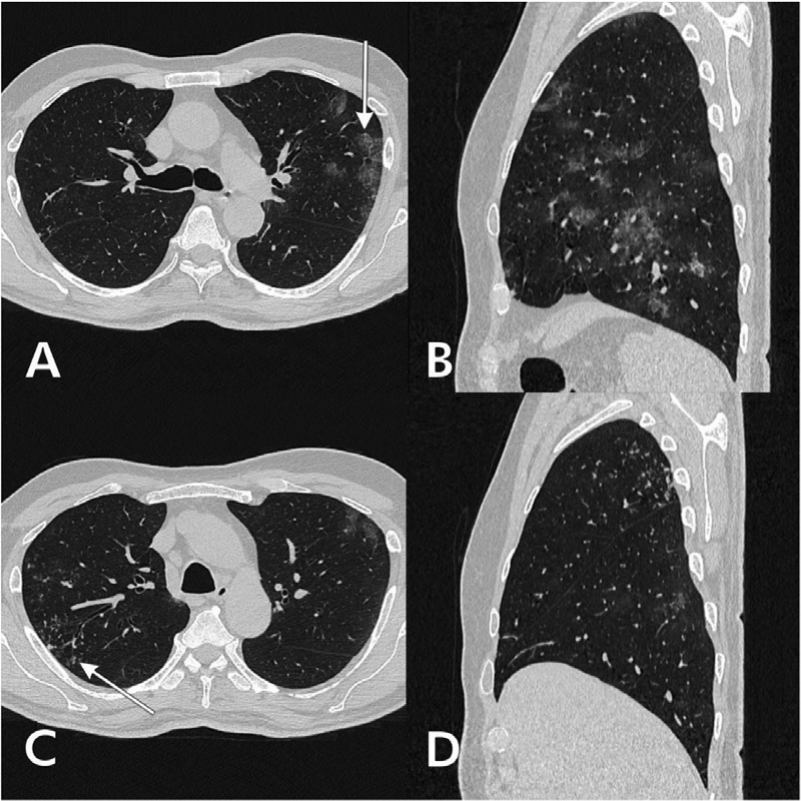
A-D: (A) Axial HRCT thorax shows rounded subpleural GGOs in left upper lobe (white arrow). (B) Sagittal HRCT thorax of the left lung shows multifocal GGOs. (C) Axial HRCT thorax shows multiple tiny nodular opacities in right upper lobe (white arrow). (D) Sagittal HRCT thorax of the right lung shows tiny nodular opacities in upper lobe and patchy GGOs in lower lobe.

HRCT thorax revealed multiple peripheral and central ground glass opacities in bilateral lung parenchyma with superimposed interstitial thickening (“crazy paving pattern”), more in left upper and lower lobes of lung (Fig. 2A,B,D). Chest CT Severity Score was 8 out of 25. Above findings were in favor of atypical pneumonia with CO-RADS (COVID-19 Reporting and Data System) category 5, typical for COVID-19.

Multiple nodular opacities were seen in right upper lobe, some of them showing “tree-in-bud” appearance (Fig. 2C,D). Fibro-bronchiectatic changes were also seen in this region. Reverse transcriptase-polymerase chain reaction (RT-PCR) for COVID-19 was positive. AFB were revealed on sputum microscopy, suggestive of concurrent TB.

Supportive therapy was initiated with oxygen supplementation for management of hypoxemia. Patient was shifted to ICU on day 5 of admission due to deterioration of symptoms, where he was intubated. However, respiratory distress failed to improve, and the patient was declared dead on day 8.

## Case 3

A 25-year-old female presented with intermittent fever and cough for 3 weeks with increasing breathlessness for 2 days. HRCT thorax & RT-PCR for COVID-19 were performed.

**Fig. 3:**
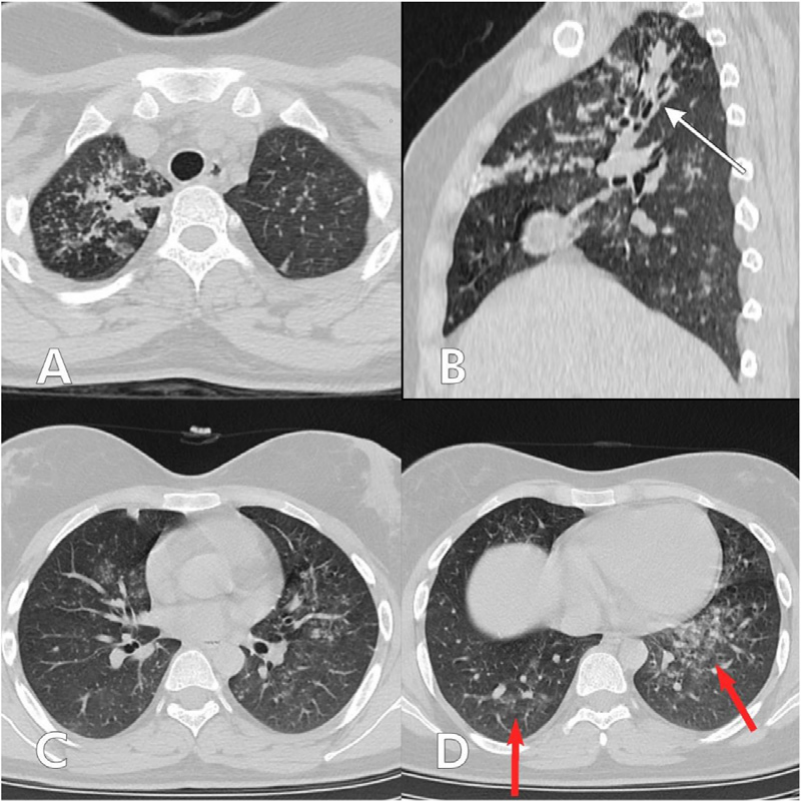
A-D: (A) Axial HRCT thorax showing tiny nodular opacities with associated patchy consolidation in apical segment of right upper lobe. (B) Sagittal HRCT thorax of the right lung showing bronchiectatic changes in upper lobe (white arrow) with associated nodules and patchy consolidation. (C) Axial HRCT thorax showing tiny nodules scattered in bilateral lung parenchyma. (D) Axial HRCT thorax showing ill-defined GGOs in bilateral lower lobes (red arrows) (Color version of figure is available online).

HRCT thorax revealed multiple tiny nodular opacities with associated patchy areas of consolidation in bilateral lung parenchyma with upper lobe predominance- more in right upper lobe (Fig. 3A,B,C). Mild bronchiectatic changes were also noted in right upper lobe (Fig. 3B). Ill-defined areas of ground glass opacification were seen in bilateral lower lobes (Fig. 3D). Initial imaging diagnosis on HRCT thorax findings was given as pulmonary TB. Sputum smear microscopy showed acid fast bacilli (AFB). However, RT-PCR for COVID-19 was positive, suggesting viral nature of the ground glass opacities in lower lobes.

Patient was commenced on first line anti-tuberculous drugs along with supportive treatment and oxygen therapy for COVID-19 infection. She was discharged after 1 week and advised to continue follow-up at the outpatient department and the Directly Observed Therapy, Short-Course (DOTS) center.

## Discussion

The 2019 Novel coronavirus disease (COVID-19) is a viral infectious disease caused by the severe acute respiratory syndrome coronavirus 2 (SARS-CoV-2) [1,2]. Meanwhile, pulmonary TB is one of the most common infective lung diseases in developing nations [12]. The concurrence of COVID-19 and pulmonary TB leads to poor outcomes [13]. Certain undiagnosed cases may come to light due to the exacerbation of respiratory symptoms caused by COVID-19. This syndemic of COVID-19 and TB may lead to severe mortality and morbidity, especially in developing nations. Furthermore, patients with pre-existing lung damage due to pulmonary TB show a worse prognosis with COVID-19 pneumonia [14]. Pre-existing lung damage may also predispose patients to COVID-19 leading to further deterioration of patient condition. Such patients with concurrence of COVID-19 and pulmonary TB can pose as a diagnostic dilemma [6]. Correct and timely diagnosis of these two co-existing entities is imperative to reduce the burden of residual fibrosis in patients who survive.

CT thorax is an essential imaging modality to identify the key features of both COVID-19 and pulmonary TB [3,5]. The classical features of pulmonary TB include mediastinal and hilar lymphadenopathy, consolidation, cavitation, pleural effusion and/or pleural thickening and miliary nodules. Findings in chronic and latent tuberculosis are characterized by bronchiectasis, lung fibrosis, fibro-cavitatory changes and ensuing architectural distortion of the lung parenchyma. Centrilobular nodules with “tree-in-bud” branching pattern are commonly seen in association with pulmonary TB [5,8,11].

The typical features of COVID-19 pneumonia comprise of bilateral peripheral ground glass opacities and or consolidation, predominantly involving the lower lobes of lung. Bilateral peripheral rounded patchy ground glass opacities may be seen in some cases. Diffuse areas of GGOs may be seen occasionally. In certain patients, GGOs may show upper lobe distribution [3,15]. Pleural thickening, bronchiectasis, lung nodules, lymphadenopathy and pleural effusion resembling pulmonary TB have also been reported [15]. Furthermore, mechanical ventilation given in cases of severe COVID-19 may incite cavitatory lesions [3,16].

Laboratory tests are key to diagnosis of both COVID-19 infection and pulmonary TB. Real time RT-PCR remains the most common and the current recommended laboratory test for the diagnosis of COVID-19 [17,18]. Rapid antigen tests may be utilized for detection of SARS-CoV-2 in individuals with high viral load [19]. Increased C-reactive protein (CRP), elevated serum ferritin, reduced white blood cell (WBC) count and lymphopenia, thrombocytopenia is frequently seen in COVID-19 patients and should be closely monitored [20].

Sputum smear microscopy for acid-fast bacilli is the fastest and one of the most indispensable investigations for detection of pulmonary TB [21]. Culture for mycobacteria is further recommended as it has a superior sensitivity than acid-fast staining [21]. Further molecular tests, DNA sequencing techniques, nucleic acid amplification methods may be used to aid diagnosis and identification [21,22].

## Conclusions

The concurrence of COVID-19 and pulmonary TB can be a diagnostic dilemma. Correct diagnosis and prompt management is imperative to reduce mortality and morbidity. Hence it is pertinent for imaging departments to identify and report these distinct entities when presenting in conjunction.

## Ethics approval and consent to participate

This study was approved from ethical committee of the institution. Written informed consent was obtained from the patients for publication of this case series and accompanying images.

## Availability of data and materials

The data and materials supporting the findings of this study are available on request from the corresponding author.

## Authors’ contributions

SSB: conceived of the study, participated in manuscript design and coordination, analyzed and interpreted the radiological studies.SSA: analyzed and interpreted the radiological studies, drafted the manuscript, participated in manuscript design and coordination. SKA: participated in manuscript design and coordination, analyzed and interpreted the microbiological studies.

The authors read and approved the final manuscript.

## Patient consent

All authors read and approved the final manuscript. Patients included in this research gave written informed consent to publish the data & materials contained within this study.

## References

[1] World Health Organization. Coronavirus. https://www.who.int/health-topics/coronavirus#tab=tab_1.Date of access June 20, 2021.

[2] Lu R, Zhao X, Li J, Niu P, Yang B, Wu H (2020). Genomic characterisation and epidemiology of 2019 novel coronavirus: implications for virus origins and receptor binding. Lancet.

[3] Kwee Thomas C., Kwee Robert M. (2020). Chest CT in COVID-19: what the radiologist needs to know. Radiographics.

[4] Jain V.K., Iyengar K.P., Samy D.A., Vaishya R. (2020). Tuberculosis in the era of COVID-19 in India. Diabetes metab syndr.

[5] Bhalla A.S., Goyal A., Guleria R., Gupta A.K. (2015). Chest tuberculosis: Radiological review and imaging recommendations. Indian J radiol imaging.

[6] Tadolini Marina, García-García José-María, Blanc François-Xavier, Borisov Sergey, Goletti Delia, Motta Ilaria (2020). On tuberculosis and COVID-19 co-infection. Eur Respir J.

[7] Tadolini Marina, Codecasa Luigi Ruffo (2020). Active tuberculosis, sequelae and COVID-19 co-infection: first cohort of 49 cases. (2020). Eur Respir J.

[8] Bomanji J.B., Gupta N., Gulati P., Das C.J. (2015). Imaging in tuberculosis. Cold Spring Harb Perspect Med.

[9] Udugama B., Kadhiresan P., Kozlowski H.N., Malekjahani A., Osborne M., Li V. (2020). Diagnosing COVID-19: the disease and tools for detection. ACS nano.

[10] Prokop Mathias, Everdingen Wouter van, Vellinga Tjalco van Rees, Ufford Henriëtte Quarles van, Stöger Lauran, Beenen Ludo (2020). Bram geurts, hester gietema, jasenko krdzalic, cornelia schaefer-prokop, bram van ginneken, monique brink, and for the covid-19 standardized reporting working group of the dutch radiological society radiology. CO-RADS: a categorical ct assessment scheme for patients suspected of having COVID-19—definition and evaluation. Radiology.

[11] Im J.G., Itoh H. (2018). Tree-in-bud pattern of pulmonary tuberculosis on thin-section ct: pathological implications. Korean J Radiol.

[12] Zaman K. (2010). Tuberculosis: a global health problem. J health.

[13] Gupta Nitesh (2020). A profile of a retrospective cohort of 22 patients of COVID-19 with active/treated tuberculosis. Eur Respir J.

[14] Digambar Behera (2020). Tuberculosis, COVID-19, and the end tuberculosis strategy in India. Lung India.

[15] Carotti Marina (2020). Chest CT features of coronavirus disease 2019 (COVID-19) pneumonia: key points for radiologists. Radiol Med (Torino).

[16] Zoumot Z., Bonilla MF., Wahla A.S. (2021). Pulmonary cavitation: an under-recognized late complication of severe COVID-19 lung disease. BMC Pulm Med.

[17] Dramé M., Tabue Teguo M., Proye E., Hequet F., Hentzien M., Kanagaratnam L. (2020). Should RT-PCR be considered a gold standard in the diagnosis of COVID-19?. J Med Virol.

[18] Scohy A., Anantharajah A., Bodéus M., Kabamba-Mukadi B., Verroken A., Rodriguez-Villalobos H. (2020). Low performance of rapid antigen detection test as frontline testing for COVID-19 diagnosis. J clin.

[19] Toptan T., Eckermann L., Pfeiffer A.E., Hoehl S., Ciesek S., Drosten C. (2021). Evaluation of a SARS-CoV-2 rapid antigen test: Potential to help reduce community spread?. J clin.

[20] Abbasi-Oshaghi E., Mirzaei F., Farahani F., Khodadadi I., Tayebinia H. (2020). Diagnosis and treatment of coronavirus disease 2019 (COVID-19): Laboratory, PCR, and chest CT imaging findings. Int J surg (London, England).

[21] Azadi D., Motallebirad T., Ghaffari K., Shojaei H. (2018). Mycobacteriosis and tuberculosis: laboratory diagnosis. Open microbiol J.

[22] Bodmer T., Ströhle A. (2012). Diagnosing pulmonary tuberculosis with the Xpert MTB/RIF test. J visual exp.

